# Should We Reject Donated Organs on Moral Grounds or Permit Allocation Using Non‐Medical Criteria?: A Qualitative Study

**DOI:** 10.1111/bioe.12169

**Published:** 2015-07-01

**Authors:** Greg Moorlock, Jonathan Ives, Simon Bramhall, Heather Draper

**Keywords:** organ donation, transplants, allocation, conditional donation

## Abstract

Conditional and directed deceased organ donations occur when donors (or often their next of kin) attempt to influence the allocation of their donated organs. This can include asking that the organs are given to or withheld from certain types of people, or that they are given to specified individuals. Donations of these types have raised ethical concerns, and have been prohibited in many countries, including the UK. In this article we report the findings from a qualitative study involving interviews with potential donors (n = 20), potential recipients (n = 9) and transplant staff (n = 11), and use these results as a springboard for further ethical commentary.

We argue that although participants favoured unconditional donation, this preference was grounded in a false distinction between ‘medical’ and ‘non‐medical’ allocation criteria. Although there are good reasons to maintain organ allocation based primarily upon the existing ‘medical’ criteria, it may be premature to reject all other potential criteria as being unacceptable. Part of participants' justification for allocating organs using ‘medical’ criteria was to make the best use of available organs and avoid wasting their potential benefit, but this can also justify accepting conditional donations in some circumstances. We draw a distinction between two types of waste – absolute and relative – and argue that accepting conditional donations may offer a balance between these forms of waste.

## Background

Organ donation worldwide has failed to meet demand for organs, and despite attempts to increase donation rates, this seems set to continue. Patients have to wait for organs to become available for transplant and many die before they are offered a suitable organ.^1^ Nonetheless, some offered organs are rejected on moral grounds. In the UK, this has included conditional donations and some directed donations. Directed deceased donations occur when a deceased person (or frequently their next‐of‐kin) agrees to donate organs only if they are allocated to a specific recipient. Conditional deceased donations occur when a person agrees to donate organs on the condition that they are allocated to (or withheld from) a specific *type* of recipient. 2

A conditional donation occurred in the UK in 1998 when a white man's next‐of‐kin specified that his organs could not be allocated to non‐whites. The organs were accepted and allocated to white people (who, by coincidence, would have been the recipients regardless of the restrictions), and several lives were saved/improved. Accepting this donation was controversial and prompted criticism in the British media.^3^ A Department of Health (DH) investigation concluded that *all* conditional deceased donation should be prohibited^4^ (this also included directed donations) and this was the position in the UK until 2010 when policy^5^ was introduced to distinguish between conditional donation and ‘requests for directed allocation’.^6^

The new policy permits requests for directed donation to recipients in ‘qualifying relationships’ (family members or close friends) to be considered, provided that:

i)  the agreement to donate is not conditional on the request being granted;
ii)  granting the request would not deprive a ‘super‐urgent’ recipient of a transplant.

A request for directed donation, unlike conditional donation, identifies a specific recipient rather than a type of person. Although this policy change raised issues which have been discussed elsewhere,^7^ it harmonized policies for deceased and living donation, where directed donation to family members or friends is commonplace.

Outside the UK, there are different approaches to conditional and directed donations. In the US, for example, an organization called LifeSharers allows people to register as donors and request that their organs are first offered to members of LifeSharers.^8^ Donation is not conditional on the request being granted, so is similar to a request for directed donation. Where it differs, however, is that potential recipients are prioritized by the donor not based on a pre‐existing relationship, but instead because they are both members of the same ‘club’.

Conditional donation and directed donation both attempt to introduce criteria into deceased organ allocation that are normally excluded. Organs from deceased donors are normally allocated according to criteria that reflect tissue matching, urgency and waiting time, and the DH report objected to conditional donations on the grounds that they prevent organs being allocated according to such established criteria.^9^

The types of conditions commonly considered in existing literature tend to be the most obviously controversial, such as those based upon race, religion or reason for illness, but it may be mistaken to therefore assume that *all* conditions are unacceptable.^10^ Refusing to accept donations with conditions attached continues to generate controversy. Although there are relatively few requests for conditional donation, the impact on donation rates of discouraging conditional donation is unclear. Some of the ethical and legal aspects of conditional donation have already received attention,^11^ and the DH 2000 report has been subject to criticism: the resulting incongruity created between living and deceased donations has been noted, for instance.^12^ There is also literature on public preferences about organ allocation more generally, which covers some of the potential allocation options that could form the basis for conditions.^13^ Some research has been conducted on public views on conditional donation,^14^ but there is a lack of in‐depth research looking not just at the views of key stakeholders, but also the justifications for these views. This article reports the findings of a qualitative study that explored conditional and directed donation with key stakeholders, and used the resulting data to inform a critical commentary on the ethical issues. The qualitative study was designed primarily to obtain data that could act as a springboard for further critical analysis, based on the premise that systematic interactions with stakeholders can help ensure the theorist is exposed to additional arguments and perspectives, leading to a thorough, nuanced and contextually aware ethical analysis; a process sometimes referred to as empirical bioethics.^15^

## Methods

Semi‐structured interviews were used, as these allow for in‐depth discussion with scope for probing and discussion on related issues. The interviews focussed on not just participants' beliefs, but also the arguments and justifications, as these were likely to provide useful data for informing philosophical reflections. A scenario‐based topic guide was used (see Table 1), comprising three scenarios and follow‐up questions. The scenarios, designed to exemplify some of the issues arising from conditional donations, focussed on desert, religious belief and immigration status.^16^ Scenarios were used to facilitate discussion because they allow participants, who are likely to be less comfortable or practised in articulating thoughts about abstract ethical principles and justifications, to begin to think about complex issues. They act as a starting point to generate discussion. When the discussion is initially focussed on concrete examples the participants can begin to articulate their thoughts in context, which then allows the interviewer to probe and explore other types of conditions and to explore underlying values and justification. Starting from a discussion of the ‘concrete’, and then using that to explore the ‘abstract’ is a more effective method then starting with discussion of abstract principles and justifications – and is a method that has been used to good effect elsewhere.^17^

**Table 1:** Interview Scenarios

*Generic Scenario Number 1* Two young men are killed in a car accident. The driver was driving recklessly and above the speed limit, which caused him to lose control of his car and crash, killing both himself and his passenger. The parents of the passenger are very distressed that their son was killed by someone acting so recklessly and are angry and upset that two lives have, in their eyes, been wasted. The parents of the passenger are very keen that their son's organs should be donated, but they have concerns about the sort of person that his organs might be transplanted to. They would like their son's organs to be transplanted to people who will look after them and live responsibly, and they consider young people to be risk‐takers who are less likely to act responsibly than slightly older people with families. They therefore request that their son's organs are only transplanted to people who are over the age of 25 and have children. In the parents' opinion, these are likely to be responsible people who will look after themselves and deserve the improvements to quality of life that an organ transplant can bring. Staff at the hospital tell the parents that they are not allowed to say who their son's organs will go to, and that decisions like this are made by medical staff on the basis of medical considerations. If the organ donation is to go ahead, the organs must be donated without any restrictions being placed on who the organs can be transplanted to. The parents decide that because of the chance that their son's organs will go to people who they consider don't deserve them, they won't give permission for their son's organs to be donated.
*Generic Scenario Number 2* A young woman dies following an accident. All of her organs are suitable for transplantation. This woman's faith and religious beliefs shaped how she chose to lead her life, and she placed great value in being what she considered a good member of her religion. Her parents know that her religious beliefs were very important to her, and are confident that, given a choice, she would have preferred for her organs to go to another member of her religion. Her parents therefore give consent for their daughter's organs to be donated, but request that priority is given to members of their daughter's religion. This request is not motivated by any dislike for a particular group of people; it is made simply because the parents believe that their daughter would prefer for her organs to go to people with similar beliefs and values to her own. The hospital staff explain that these sorts of requests cannot be complied with under existing policy, which states that organs will be given to the people who are in greatest need and who are the best match.
*Generic Scenario Number 3* A man has died in an accident. All of his organs are suitable for donation, and his family are very keen that his organs should be donated. Prior to his death, the man had expressed concerns about the number of immigrants that had moved to his area. He believed that a lot of the immigrants were taking jobs, benefits and resources that would otherwise be given to local people. He believed that people like himself who have lived in the area all of their lives should have everything that they need before things are given away to people who have arrived from other countries. The man had made clear, prior to his death, that if he died he wanted to donate his organs, but that he didn't want his organs to be transplanted to immigrants. Knowing that their relative held these views, the next‐of‐kin agree to donation only if the organs do not go to immigrants. Staff at the hospital tell the next‐of‐kin that they are not allowed to say who the organs should or should not go to, and that if the organs are going to be donated they have to be donated to the general pool. The family insist that they will not agree to donate the man's organs if there is a risk of them going to immigrants, as the deceased man would have been opposed to this.

Forty participants were recruited from three stakeholder groups: transplant staff, potential organ recipients and potential organ donors. Staff participants were recruited from the liver and kidney units of a large UK transplant centre. Staff with a range of transplant roles were recruited (see Table 2). Potential organ recipients were defined as patients on a transplant waiting list or who were likely to be listed in the foreseeable future. All were patients at the same UK transplant centre. The potential donor category included members of the healthy general population, since anyone can potentially donate organs. Because South Asian (SA) donation rates in the UK are relatively low,^18^ we were interested to explore the idea that conditional donation has potential to increase organ donation in the local SA population. Accordingly, we attempted to ensure that our sample of potential donors comprised a good proportion of SA participants (up to half), to increase the possibility that we might learn something about this.

**Table 2:** Outline demographics of participants

	Acronym	Number	Gender	Other Information
Transplant Staff	TS	11	Not specified for staff, because it is potentially identifying information	2 × Consultants Anaesthetists/Intensivists 3 × Specialist Nurses – Organ Donation 2 × Liver Recipient Transplant Co‐ordinators 2 × Liver Transplant Surgeons 1 × Liver Consultant Physician 1 × Clinical Nurse Specialist in Addiction Psychiatry
Potential Donors	PD	20	13 female, 7 male	10 × South Asian ethnicity 10 × any other ethnicity
Potential Recipients	PR	9	4 female, 5 male	4 × liver, 5 × kidney

Each participant gave written informed consent before their interview, and each interview was audio‐recorded and transcribed verbatim. A favourable opinion was given by an NHS research ethics committee (Ref. 10/H1208/34) and the University of Birmingham research ethics committee (Ref. ERN_10‐0900), and appropriate R&D permissions were obtained.

The interviews were analysed by Moorlock using NVivo software. ‘Initial Coding’ as described by Saldana^19^ was undertaken using a relatively open‐ended approach. As more codes were introduced, these were sorted into themes. Data were coded, and themes developed, with a view to identifying reasons for and against conditional and directed donation (both practical and theoretical), which is in keeping with the aim of the empirical research being to expose the theorist to additional arguments and perspectives. The codes and themes developed as the project progressed, and earlier transcripts were re‐examined to check for instances of newer codes. A sample of transcripts was independently coded by Draper and Ives to check consistency of coding and to try to minimize researcher bias.

## Results

The collected data was rich and covered many ethical issues arising from conditional donation. The results presented here are underpinned by a common theme: the use of medical criteria to allocate organs. Some interpretation is included alongside the narrative of the results in order to make clearer the meaning of specific quotations.

### 1. Organs should be allocated using medical criteria

Conditional/directed donation can disrupt the usual processes of organ allocation. The scenarios therefore prompted more general discussion of how organs *ought* to be allocated. Participants across all categories generally felt that organs should preferably be donated unconditionally, primarily because this allows for allocation according to medical criteria. Participant PD6 cast doubt on the public's ability to make good decisions in the context of organ allocation. That this participant thought that scientific data should be providing the basis for good allocation decisions points to an assumption that medical criteria are better because they are objective, not biased by personal opinion:

They should leave that type of judgment for scientists who have the data to back up those kinds of claims … they should be guided by numbers and science … and not opinion. People [the public] can't be trusted to make good decisions, I think we all know that – PD6

The view that medical criteria are more substantial than opinion was shared by many participants across all groups. Medical criteria were often regarded as facts that produce an allocation process that avoids judgments involving complex/controversial values.

Criteria such as ‘greatest need’, ‘likely transplant outcomes’ or ‘best match’ were all regarded as ‘medical’:

I still think that the decision should be made medically on perhaps who is less likely to reject it and who is more … likely to come out of it successfully. – PD1

what's their chances of survival, what's the chances of the graft surviving, as long as you know a reasonable length of time and they're then allocated onto the list in that way – TS5

### 2. Justifications for using medical criteria

#### Objectivity

A reason frequently given for preferring medical criteria was that they remove emotion from sensitive situations and exclude individuals' biases from allocation decisions. PD2 illustrates the sometimes tacit assumption that it is undesirable for organ allocation to be based upon the views of someone emotionally involved in the situation:

Yes I do, I really do … you've gotta take emotion out of the argument and I think that's the only way to do it, is try and get someone who hopefully has a more objective view than the family of the deceased. – PD2

Another potential donor pointed to the avoidance of bias and suggests that the medical community should be focussing on medical need rather than other (potentially still relevant) criteria such as dessert:

I'm hoping the medical community will make them without bias and on medical needs, somebody who needs an organ in order to survive or a better quality of life, rather than somebody's more deserving – PD28

#### Consistency and fairness

All participants thought that allocating organs fairly was important, and most felt that allocating according to medical criteria was the best way to achieve this. For example, one member of staff (TS4) favoured medical criteria, and argued that their objectivity helped to justify allocation decisions to others. This suggests that the criteria contribute to robust and defensible allocation, so that allocation is not just fair, but also *seen* to be fair:

as long as the principles on which you do the allocation is transparent, and it's objective, so that you can justify why you give it to A or B or why C doesn't get a chance at getting the organ, I think that's the best you can do – TS4

The importance of organ allocation being fair and transparent seemed to be emphasized because the stakes are so high: sometimes literally life or death. There was a feeling that when stakes are lower there might be room to listen to individual preferences:

I think it's mainly because of it's that life and death thing isn't it? As opposed to just cash or whatever. Just giving stuff away … you could give it to anybody couldn't you? Him on the street if you wanted. But when it comes to a matter of life and death it's a different issue. – PR2

Although fairness was important to all participants, there were individual cases of disagreement over which criteria were relevant to a fair decision. The most commonly held view was that only criteria related to outcomes with or without a transplant are relevant:

Ok, to me fair would be based on like I say if you have your waiting list, you have an organ come in, I think it should all be based on effectively percentage chance of … of survival's the wrong word but being able to lead a reasonable life afterwards. – PD9

Some participants considered whether fair allocation should include why someone needs a transplant, which could be construed as a departure from the usual medical allocation criteria. Of these participants, many thought that whilst a patient causing their own illness might make them less deserving of a transplant, this should nonetheless be excluded from allocation decisions. The potential donor below, who felt that deservingness should be considered, was in a small minority:

Fair is I don't know what I've already mentioned, like based on how the person and why the person requires the organ in the first place. – PD7

There was also one participant who questioned the meaning of ‘greatest need’. Most participants regarded medical need as absolute and fact‐based, but one member of transplant staff doubted the objectiveness of this criterion, and appreciated that need may be contingent upon a number of factors, some of which may not be medical:

Greatest need by whose definition, that's my problem. Who's defined the greatest need, you know, what because they're hooked onto a hundred million life support machines, you know what about the greatest need of someone's gonna be left without a husband, so you know, once again it's been by definition. What we all interpret as greatest need is different for us all. – TS6

#### Best way to fulfil goal of transplantation

A view prominent across all groups was that organ allocation should not be based on who is more worthy to live, because the goal of transplantation should be simply to restore health. It therefore followed that transplant staff should not be considering factors beyond the ‘medical’ when allocating organs, as these considerations were nothing to do with the goals of transplantation. This participant summed up the view succinctly:

[treat] everybody as if they're the same and … other factors should just be completely ignored, all they're really there to do is to make people healthy regardless of any of their history. – PD4

This view suggests that the purpose of transplantation is to meet the medical needs of those who require transplants, and nothing more. The following participant, although mentioning classes of people, again links this back to the idea of transplantation meeting needs of people requiring transplants:

I think it's up to the medical staff to actually decide who gets that organ. … Because I think they have a better idea of who's out there and which class of people actually need a liver transplant – PD24

### 3. Acceptable deviation from medical criteria

Despite widespread support for medical criteria being the best basis for allocation, there was some support for limited types of conditional or directed donation. Several participants felt that directed donation to family members or close friends was acceptable. Some drew parallels to living donation where this is commonplace, whereas others highlighted the fact that people display levels of partiality for their loved ones in every‐day life, and that this is often considered acceptable:

I think that extends in to every … aspect of your life, you know lots of things you do for family and loved ones that you would never ever do for random people and I don't think that's a bad thing on the whole. – PD1

There was also some support in all groups for directed donations to children. This was most often justified on the grounds of children's perceived vulnerability, or that they have lived less life than adults:

Just because a child has I don't know, they've arguably probably got more of their life to lead, they have had less of a life because they're younger than you know someone older and I don't know I guess they'd be seen as being more vulnerable, weaker, which is not necessarily true, but they've just got more of their life to live, haven't they? – PD5

### 4. Unacceptable forms of conditional donation

Although inclined to reject bypassing medical criteria for allocation, many participants felt uneasy about the cost of turning down potential donations. For these participants, saving lives carried more weight than strict adherence to medical criteria:

at the end of the day it's better to save some than to throw a chance away that, you know I mean, people could last another 10, 20 years with the treatment and what you have … so it's giving 5 to 6 people a chance to live that little bit longer in life and probably enjoy their lives – PR4

For many participants (mostly in the recipient and donor groups), the consequence of saving additional lives could justify the acceptance of conditions that would otherwise be considered unacceptable, including the racist conditions in the 1998 case:

Well the people who were you know judging this basically thought well you know, we'd rather that it went to the people than we just flat out refused, so I think they were probably in the right there. – PD7 (discussing the 1998 conditional donation)

Many participants thought that accepting conditions should be a last resort, justified only if the alternative was to turn down a donation, because refusing a donation is a waste of a potentially life‐saving resource:

I think that your default position should be on medical needs and only if they are risking to lose organs, then maybe as a kind of sub parameter, perhaps a back door, we should allow the donor actually to voice conditions or direct the donation. – TS10

### 5. Staff concerns about integrity of the transplantation system

In contrast to some of the non‐staff participants, many (although not all) of the staff participants viewed turning down conditional donations as unfortunate but necessary in order to maintain the integrity of the transplantation system:

Our society at the moment is prepared to pay the price of losing the occasional organ in this situation because of the, you know, the greater good and the overriding principle really – TS3

### 6. Limitations

Although the study specifically attempted to ensure that SA potential donors were well represented, no noticeable differences were found between the views of SA potential donors and donors of other ethnicities. Given the known differences in attitudes towards organ donation^20^ from this population and the general population, this was surprising. The recruitment methods necessary to secure sufficient participants within the time constraints of the study will have led to a non‐representative sample – although it is worth noting that the aim of this kind of empirical study is not to obtain representativeness, but to explore personal accounts and generate ideas. Initial recruitment attempts were made through an inner‐city General Practice, with invitation packs being sent in the post to 100 SA potential donors. This yielded no responses. As a secondary recruitment method, SA participants were recruited via a combination of University of Birmingham advertising in a weekly e‐newsletter and snowballing (asking colleagues, friends and existing participants to pass on information about the research to people who may be interested in participating). This resulted in the SA sample containing participants who were generally well educated, who had links to the research community at the University, and were generally pro‐organ donation.

Transplant staff and potential recipients were mostly from the Liver or Kidney Units, and different organs may raise different issues. The heart, for instance, is often noted for its symbolism^21^ and this may impact upon people's views. Further research with staff/patients relating to other organs may therefore be beneficial.

## Discussion

Our data illustrate the dilemma that conditional and directed donations pose: although the conditions themselves may be objectionable or require deviation from preferred, national allocation criteria, an offered organ is still a life‐saving/life‐improving resource, and refusing this resource on ideological grounds has potentially lethal consequences for those awaiting transplantation. The following discussion will therefore assess the robustness of the ideological grounds espoused by participants, to establish whether they provide compelling reasons to exclude non‐medical criteria and turn down conditional or directed donations.

### The privileged position of medical criteria

Participants' views on the right way to allocate organs significantly influenced how they viewed conditional and directed donations. The preference for organs to be allocated according to medical criteria broadly reflects how organs are currently allocated, and our findings here are in‐line with other studies,^22^ where participants also favoured adherence to medical criteria. This preference meant that all conditional donations were regarded as non‐ideal. Presented with this view, it could be tempting simply to reject conditional and directed donations as contravening the principles of allocation important to stakeholders. Further analysis, however, will highlight:

i)  problems in how participants idealized medical criteria, undermining the view that medical criteria provide an objective and undisputable basis for organ allocation.
ii)  how participants' justification for using medical criteria can also support deviation from the same criteria in specific circumstances

### The differences between medical and moral criteria

Medical criteria^23^ were considered to include greatest need, urgency, best match and predicted transplant outcomes, and there is some overlap between these criteria: for instance, urgency may be related to many conceptions of greatest need, and best tissue match is related to predicted outcomes. Criteria that were frequently rejected as being non‐medical included race,^24^ religion, or social value/utility, but also those that are arguably medically‐related, such as disease type or cause of illness. The perceived differences between these medical and non‐medical criteria are potentially complex. Merely making reference to some aspect of a patient's medical condition did not render a criterion medical in the eyes of participants – otherwise, cause of illness would be considered medical. Instead it seems that participants conceived of medical criteria as being both health‐focussed and forward‐looking, insofar as they play a role in answering the question ‘what would happen to this patient's health if they did/did not receive these organs?’ This is a very narrow view of what is relevant when allocating organs, but reflected a view about the goals of transplantation, which will be discussed in more detail later.

There was widespread belief that depending solely upon medical criteria results in the right allocation of organs, but the justification of this position was commonly grounded in the assumption that medical criteria provide an unambiguously objective allocation process. Veatch has already noted, however, that such criteria are not objective, and that moral argument is required both to define these criteria and determine how they ought to be balanced against one another.^25^ For example, many participants thought that organs ought to be allocated according to greatest need, but did not recognize that ‘greatest need’ is a complex concept that involves balancing concerns such as urgency, current quality of life and potential to benefit from a transplant. Even the superficially more simplistic criterion of ‘urgency’ requires consideration of how the urgent risk of death should be balanced against the urgent need to improve quality of life.

Failing to recognize the complexity of these criteria often resulted in participants assigning extra weight to them and giving them an elevated status amongst other possible criteria. Allocation based upon these medical criteria is no more ‘matter of fact’ than allocation based upon a person's previous behaviour, their race or their religious beliefs, so appealing to medical criteria as the correct basis for organ allocation on the grounds of their supposed objectivity is unconvincing. Furthermore, the overt valuing of ‘objectivity’ is itself arguably subjective. The assumption that organ allocation can be made without subjectivity or the application of values is, therefore, deeply problematic.

### Are medical criteria the only relevant criteria?

Although participants may be mistaken in thinking that medical criteria are an especially privileged category of allocation criteria, they did suggest other reasons to maintain organ allocation based upon these criteria. For instance, participants generally felt it important that organ allocation should be just and fair, which in simple terms requires that only differences that are *relevant* to the situation in hand are considered when choosing one patient over another. There was agreement from participants that some criteria are irrelevant to organ allocation and are based upon flawed reasoning or prejudice. These included criteria arbitrarily based upon religion or sexuality (which, one presumes, will normally tend to lack independent robust justification as the basis for allocation criteria – and as such are no more a suitable way of differentiating between patients than considering what their favourite television programme is). Basic principles of justice and fairness provide strong arguments against irrelevant criteria such as these being routinely used to allocate organs.^26^ Participants also appeared, however, to have a more restricted account of what is relevant to organ allocation decisions (what we will refer to as ‘medically relevant’) than a more theoretical approach might initially suggest (we will refer to this broader set of criteria as being ‘morally relevant’). All things that are medically relevant are morally relevant, but not all morally relevant considerations were thought to be medically relevant.

The difference between medically and morally relevant can be illustrated by considering the criterion of ‘predicted outcomes’, which could be defined as a primarily medical criterion. A liver transplant might give one patient a 90% probability of five year survival, or an alternative patient a 10% probability. The answer to the question of which has the best predicted outcome is clearly the first patient. One may reasonably ask, however, why five year survival is the relevant outcome. If outcomes are important, then why define the relevant outcome so narrowly? Outcomes could also include, for instance, what the recipient is likely to do with their life post‐transplant. If one patient was expected to save many lives, and the other expected to cause much suffering to people, then, all other things being equal, there is a strong moral argument in favour of choosing the former as this would bring about the most good from the available options. A less abstract example might be that the patient with 90% chance of five year survival will live, but with a relatively poor quality of life. In contrast, the patient with a 10% chance of survival would live, if at all, with a very good quality of life. It is not obvious that a good chance of a poor quality is preferable to poor chance at a good quality of life. Participants seemed to think, however, that factors beyond the narrow medical conception of outcomes are not appropriate considerations for organ allocation (and so are not medically relevant), even though they could be the sorts of considerations that are *morally* relevant. There were two main ways in which participants attempted to justify this position:

i)  Donors/Transplant staff are poor judges, and any judgments would not be sufficiently robust – there is too much room for uncertainty in factors like social value or quality of life and therefore such factors increase risk of arbitrary injustice.
ii)  Donors/Transplant staff ought not to judge – such factors are irrelevant to the goals of transplantation and medicine.

The first point is partly an empirical claim, but is intuitively reasonable. While medical staff are well‐positioned to assess a tissue type, current state of health and likely future state of health, they are not generally uniquely well positioned to establish, for example, how deserving a patient may be, or where ultimate responsibility for their illness lies. This uncertainty argument is not straight‐forward, however, because there is also significant uncertainty in predicting even the narrowly defined (see earlier discussion on predicted outcomes) medical outcomes of transplantation, yet this is still considered to be an acceptable means of choosing recipients. It may be that the extent of uncertainty is greater when it comes to non‐medical criteria, but this will not always be the case, so does not provide a strong reason to exclude wider morally relevant considerations.

The second justification presents an interesting problem: although many participants felt that other considerations, such as responsibility for illness or social worth may be morally relevant, they also felt that it would be wrong for them to feature in the allocation process. Some explanation is required of why *morally relevant* considerations should be excluded.

Participants' justification tended to employ the distinction between medical and moral criteria we have just challenged. Specifically they thought that medical criteria are robust and objectively defensible whereas moral criteria are matters of opinion and open to disagreement. This view is also endorsed by the Organ Procurement and Transplantation Network (OPTN) in the US, who state that: ‘in public policy related to allocation of organs there is a widespread consensus that certain social aspects of utility should not be taken into account’.^27^ OPTN's justifications for this are that an individual's social worth, for instance, is a matter of opinion or a matter of good fortune in the natural lottery,^28^ but this is unconvincing.^29^ The claim that these are matters of opinion draws on the same false distinction between medical and non‐medical criteria employed by our participants. Although one may have an opinion on someone's social worth that is grounded solely in unjustifiable prejudice, it is also possible for there to be defensible accounts of social worth that are as objective as medical criteria. The latter point about natural lotteries may be correct, but applies equally to medical criteria (one patient may fortunately have excellent expected outcomes, whereas another may unfortunately have too many co‐morbidities to be sufficiently likely to survive, and this may be purely a result of chance) so does not provide an absolute reason to exclude non‐medical but still morally relevant criteria. The views of participants went slightly beyond the arguments espoused by OPTN: they also centred on the idea that transplantation services exist to meet the medical needs of those who require transplants, and that wider considerations are beyond the scope of transplantation goals.

### The advantages of medical criteria

Although it has been argued that medical criteria do not provide the objective basis for allocation that is often assumed, and that the distinction between the medical and the moral is not clear‐cut, there remain reasons to prefer medical criteria as the general basis for organ allocation. Primarily, they allow for a balance of life‐saving, life‐improving and equal treatment to be struck, which allows the needs of transplant recipients (and society more generally) to be met. For unconditional donations, at least, this balance can be considered to be the optimal outcome of organ allocation. This assumes, of course, that the current UK allocation system is based on the best empirical evidence and careful reasoning to ensure that competing considerations are appropriately balanced. This does not equate to a claim that the allocation system is perfect. It may instead be the best currently available attempt at balancing competing considerations in genuinely dilemmatic situations. If this is the case, then unnecessary deviation from current allocation policy would be undesirable because deviation is unlikely to result in the optimal outcomes, which would be a waste of the potential benefits provided by donated organs. This use of potential benefits and avoidance of waste would therefore provide a good prima facie reason to base organ allocation on primarily medical criteria as a general rule, but does not itself provide a reason to rigidly exclude non‐medical criteria, especially if doing so might *reduce* waste and improve outcomes in particular instances. Discussion will now briefly consider reasons that were posited by participants for deviating from strict adherence to medical criteria.

### Sometimes non‐medical criteria can be acceptable

Although participants generally regarded conditional donations and their associated introduction of non‐medical criteria into allocation as non‐ideal, there were some specific conditions or directions that were considered by many to be acceptable deviations from medical criteria. For instance, some participants thought it acceptable to direct a donation to a family member or towards (non‐specified) children more generally. In a survey by Neuberger and Mayer, 36% of respondents supported directed donation to family members, and 59% supported directed donations to children.^30^ Neither was permissible following the DH's 2000 report, although the March 2010 revision now allows directed donation to family members.

Participants' views broadly accorded with current guidance on requests for directed donation, although participants tended to feel that these directions could be more than just requests, so it would be permissible for the donation to be contingent on the request being granted. This is in line with living organ donation where donors in qualifying relationships are able to specify that they *only* want to donate their kidney or liver lobe *if* it is transplanted to a specific person.^31^ The DH report concluded that all conditional donations were unacceptable because they violate the fundamental principles that organs must be donated altruistically and allocated according to greatest need; and it is unclear how a request for a directed donation would not also violate these principles. The March 2010 guidance appears to acknowledge that it is acceptable for a donation to be allocated contrary to greatest need (i.e. it can be directed to a family member, who may not be the person in greatest need). However, that guidance also states that ‘[c]onditionality offends against the fundamental principle that organs are donated voluntarily and freely and should go to patients according to the agreed criteria’.^32^ This statement is confusing; whether someone is freely and voluntarily deciding to donate is entirely unrelated to that person placing restrictions on who can receive their organs. Such restrictions can stop organs being allocated according to agreed criteria, but then so could a (now acceptable) request for a directed donation. It seems that the March 2010 guidance represents an attempt to meet the needs/wishes of the next‐of‐kin and the donor, whilst also allowing for the need to save the lives of the most urgent recipients to be met, but in doing so it has introduced further inconsistency. The legal grounding of this guidance has been criticized elsewhere,^33^ but the position it proposes also appears to lack sufficient ethical justification. The needs of the most urgent patients are allowed to trump the known preferences of the next‐of‐kin/donor in deceased donation, yet this is not true for living donation. It is not obvious why the donor being dead is a relevant difference.^34^ This confusing situation could easily be resolved by making requests for directed donation akin to living donation, where the donation is understood to be contingent on the request being granted.

### Wasting Potential

Some conditions or directions were regarded by participants to be acceptable, such as those prioritizing family members or children, although many also thought that unconditional donation was preferable. Other conditions were regarded as being irrelevant to organ allocation, unjustifiable and plainly wrong; those, for instance, involving race or sexuality. Despite this, many participants from all groups felt that even objectionable conditional donations should be accepted if the alternative was to refuse an organ that was life‐saving/improving. Whilst this view initially appears to be at odds with favouring allocation based on medical criteria, an argument can, and will now, be made for endorsing a general reliance on medical criteria but also permitting exceptions.

Earlier discussion introduced the idea of meeting need, and argued that a general allocation policy based upon medical criteria will meet the needs of recipients relatively effectively. Waste occurs when need is not met as fully as it could be, and avoiding waste is important given the organ shortage. A pertinent distinction can be drawn between two types of waste: absolute and relative. Rejecting a conditional donation outright is an example of absolute waste: a donation and all its potential benefit is lost. Accepting a conditional donation, however, may result in a form of *relative* waste, particularly if it sets a precedent for future conditional donations.

Relative waste can be defined as the difference between the maximum benefit that an available organ could provide, and the actual benefit that it does provide. In the UK, organs are not allocated *solely* to maximize benefit to individual patients, but are instead allocated in order to balance potentially competing considerations of utility and justice, which helps to maximize benefit to society. It is this overall benefit that should be considered here. When organs are donated unconditionally, they can be allocated according to the current criteria which allows for the optimal overall benefit to be extracted from them. A donation with conditions or directions attached changes the choice landscape: the usual optimal benefit is no longer available,^35^ so the choice becomes one of obtaining sub‐optimal benefit or no benefit. By allowing conditions placed by donors to influence allocation, however, additional criteria would enter the equation which would likely compromise the overall benefit provided (compared with the same organs donated unconditionally). Conditional and directed donations therefore have the potential to increase relative waste.

This presents a situation that requires careful balancing: accepting conditional or directed donations, as suggested by many participants, can avoid the absolute waste of turning away organs, but may result in increased relative waste. Isolated instances of accepting conditional or directed donations could reduce overall waste (as organs would be accepted that otherwise would not be), but the prospect of these donations becoming more widespread (and the potential for donations that previously would have been made unconditionally now being made conditionally) could lead to increases in relative waste outweighing any other gains. For instance, if an increased number of conditional donations led to reduced public support for the transplantation system, donation rates might subsequently drop, and the number of transplants would therefore be reduced, thereby reducing overall benefit.

This was something that many staff participants were particularly wary of. They believed that organ donation relies upon the goodwill of the public, and that this goodwill may require an allocation system that people can trust. The potential donors who participated in this study did not share these concerns, but larger‐scale research would clearly need to be conducted to establish the likely impact upon donation rates. Minimizing waste will increase the good that can be achieved by transplantation. It is important, however, that potentially competing types of waste are balanced in a way that results in greatest benefit. Achieving this balance is complicated, and there are many uncertainties involved, but to turn down all conditional donations is not obviously the correct approach.

## Conclusions

Given that participants thought that medical criteria ought to generally be used to allocate organs, it follows that conditional donations were viewed as non‐ideal, but the discussion of conditional donation has highlighted several points of wider interest for organ allocation. It has been argued, for instance, that participants' views on the nature of medical criteria were often mistaken, and do not provide reasons to exclude all of the criteria that participants viewed as non‐medical. In addition, it has been argued that participants held a narrow view of what is medically relevant (and therefore considered relevant for organ allocation), which often tended to exclude considerations that could be considered morally relevant. This led to the somewhat problematic position from participants that some morally relevant considerations ought to be excluded from organ allocation.

Moreover, and specifically in relation to conditional and directed donations, it has been argued that a convincing justification for allocating organs primarily according to medically relevant criteria – the avoidance of waste – also provides a reason to consider other criteria when the alternative is to turn down an offer of organs. This then gives rise to a complex situation requiring the balance of potentially competing forms of waste.

It would be wrong to conclude solely on the basis of the findings and arguments presented in this article that conditional donations ought to be accepted. Issues such as maintaining public trust in the allocation system, involving publically funded organizations in discriminatory practices, and the potential for furthering broader healthcare inequalities are also important and have not been considered here. But such issues must be considered against the arguments put forward by our participants and advanced in this paper (and others)^36^ that turning down conditional donations may be wasting potentially life‐saving resources.

## Footnotes

1

NHS Blood and Transplant . 2014 
*Organ Donation and Transplantation Activity Figures*
. [cited 2015 Feb 5] Available from: http://nhsbtmediaservices.blob.core.windows.net/organ‐donation‐assets/pdfs/annual_stats.pdf [cited 2015 Feb 5].

2

A.J. Cronin & D. Price , Directed Organ Donation: Is the Donor the Owner?, Clinical Ethics 2008; 3: 127–131.2089046210.1258/ce.2008.008018PMC2948558

3

BBC News , 2000 
*Racist Organ Donation Condemned*
. [cited 2015 Feb 5] Available from: http://news.bbc.co.uk/1/hi/health/652132.stm.

4

Department of Health . 2000 
*An Investigation into Conditional Organ Donation*
. [cited 2015 Feb 5] Available from: http://webarchive.nationalarchives.gov.uk/20130107105354/http://www.dh.gov.uk/prod_consum_dh/groups/dh_digitalassets/@dh/@en/documents/digitalasset/dh_4035465.pdf.

5

NHS Blood and Transplant . 2010 
*Requested Allocation of a Deceased Donor Organ*
. [cited 2015 Feb 5] Available from: www.nhsbt.nhs.uk/to2020/resources/finalguidanceen.doc.

6
Although the new policy uses the term ‘requests for directed allocation’, the term ‘request for directed donation’ will be used throughout this article to avoid confusion.

7

J.F. Douglas & A.J. Cronin . Requested Allocation of a Deceased Donor Organ: Laws and Misconceptions. J Med Ethics 2010; 36: 321.2051135310.1136/jme.2010.037135

8

Lifesharers , 2015 
*Lifesharers Homepage*
. [cited 2015 March 9] Available from: http://www.lifesharers.org.

9

Department of Health . *op. cit.* note 4.

10

D. Price . Conditional Organ Donation – A View of the Ethicist, Transplantation 2008; 85: 1540–1541.1855105410.1097/TP.0b013e318172d9c3

11

T.M. Wilkinson . Racist Organ Donors and Saving Lives, Bioethics 2007; 21: 63–74;1784549010.1111/j.1467-8519.2007.00526.x G. Pennings . Directed Organ Donation: Discrimination or Autonomy? J Appl Philos 2007; 24: 41–49; R.M. Veatch . Transplantation Ethics. Washington, DC: Georgetown University Press; 2000; A.J. Cronin & J.F. Douglas . Directed and Conditional Deceased Donor Organ Donations: Laws and Misconceptions. Med Law Rev 2010; 18: 275–301;2073943510.1093/medlaw/fwq019PMC2927926 Cronin & Price . *op. cit* note 2.

12

Cronin & Douglas . *op. cit* note 11.

13

C.J. Browning & S.A. Thomas . Community Values and Preferences in Transplantation Organ Allocation Decisions. Social Science and Medicine 2001; 52: 853–861.1123486010.1016/s0277-9536(00)00187-8

14

J. Neuberger & D. Mayer . Conditional Organ Donation‐the Views of the UK General Public Findings of an Ipsos‐Mori Poll'. Transplantation 2008; 85: 1545–1547.1855105610.1097/TP.0b013e318172db8a

15

J. Ives & H. Draper . Appropriate methodologies for empirical bioethics: it's all relative. Bioethics 2009; 23: 249–258.1933852510.1111/j.1467-8519.2009.01715.x

16
The scenarios were not built around the conditions already frequently discussed in the literature, such as conditional donation excluding alcoholics, due to concerns about the impact that this may have on participants from the recipient group whose health conditions were associated with their previous lifestyle choices. At the same time, the scenarios needed to be sufficiently controversial to generate discussion of the rights and wrongs of imposing conditions. It was therefore decided to frame the scenarios in more general terms to avoid distressing participants. The local research ethics committee requested that the scenarios were made even more general e.g. not to make reference to any specific religion.

17
See, for example: J. Ives , H. Draper , H. Pattison & C. CWilliams . Becoming a father/refusing fatherhood: An empirical bioethics approach to paternal responsibilities and rights. Clin Ethics 2008; 3: 75–84.

18

NHS Blood and Transplant . 
*South Asian Campaign*
. [cited 2015 Feb 5] Available at: http://www.organdonation.nhs.uk/campaigns/other_campaigns/detail.asp?id=3.

19

J. Saldana . The Coding Manual for Qualitative Researchers. London: SAGE Publications; 2013 81.

20

F.S. Al Khawari , G. Stimson & A. Warrens . Attitudes Toward Transplantation in UK Muslim Indo‐Asians in West London. Am J Transplant 2005; 5: 1326–1331.1588803710.1111/j.1600-6143.2005.00814.x

21

M.L.A. Sadala & A.G.S. Noedir . Heart transplantation experiences: a phenomenological approach. J Clin Nurs 2008; 17: 217–225.1857879710.1111/j.1365-2702.2007.02206.x

22

A. Tong et al. Community Preferences for the Allocation of Solid Organs for Transplantation: A Systematic Review. Transplantation 2010; 89: 796–805.2009057010.1097/TP.0b013e3181cf1ee1

23
Although we argue that the distinction between medical and non‐medical criteria is not clear‐cut, for simplicity's sake we will use these terms to discuss various criteria as this is how they were grouped by participants.

24
It should be noted that sometimes race *is* medically relevant insofar as it is linked to things that are medically relevant, such as HLA typing to assess compatibility between donor and recipient.

25

Veatch , *op. cit.* note 11.

26
Although justice provides reasons against these types of criteria, it is important to note that other reasons may outweigh simple justice concerns.

27

Organ Procurement and Transplantation Network . Ethical Principles to be Considered in the Allocation of Human Organs. [cited 2015 Feb 5] Available from: http://optn.transplant.hrsa.gov/resources/bioethics.asp?index=10.

28
Ibid.

29
We take ‘natural lottery’ to refer to the Rawlsian idea of social and natural lotteries, suggesting that there are some things that people are born with, or into (e.g. economic situations), that are the result of chance, so any advantage or disadvantage conferred to them throughout life as a result of these things is, in one sense, undeserved. Someone born into a wealthy family may be able to achieve greater social worth than someone born into extreme poverty, for instance.

30

Neuberger & Mayer , *op. cit* note 14.

31
In practice, living donors may be willing for their donated organ to be transplanted to a stranger if, after it has been removed, it turns out not to be suitable for the originally intended recipient. They are also given the options of having the organ re‐transplanted to themselves, for the organ to be donated for research, or for the organ to be disposed of. On the other hand, living donors who do not have a specific recipient in mind are not permitted to make any stipulations or conditions on their donation.

32

NHS Blood and Transplant . *op. cit* note 5.

33

Douglas & Cronin . *op. cit.* note 7.

34
And if it is a relevant difference, then it may raise interesting questions about whether the needs of the living can outweigh the wishes of the dead in more cases.

35
We assume that it would be unacceptable to agree to the conditions placed on a donation and then ignore them when allocating the organs.

36

T.M. Wilkinson , *op. cit.* note 11.

